# The significance of internal calcifications on perinatal post-mortem radiographs

**DOI:** 10.1016/j.crad.2020.03.007

**Published:** 2020-07

**Authors:** C. Reid, O.J. Arthurs, A.D. Calder, N.J. Sebire, S.C. Shelmerdine

**Affiliations:** a Great Ormond Street Hospital for Children NHS Foundation Trust, London WC1N 3JH, UK; b UCL Great Ormond Street Institute of Child Health, London, UK; c NIHR Great Ormond Street Hospital Biomedical Research Centre, London, UK

## Abstract

**AIM:**

To determine whether the presence of internal calcifications on perinatal post-mortem skeletal surveys (PMSS) are associated with certain diagnoses of fetal loss.

**METHODS AND MATERIALS:**

A 6-month retrospective, single-centre, cohort study was conducted on PMSS performed for perinatal death assessment. One reader re-reviewed all PMSS images for the presence and location of internal calcifications, and noted whether these were included within the original radiology report. Findings at autopsy were then reviewed independently by a second researcher and cause of fetal loss or main diagnosis recorded. Chi-squared tests were conducted to identify differences between those with and without internal calcifications at PMSS.

**RESULTS:**

Two hundred and thirty perinatal deaths (mean gestational age 18 weeks; average 12–35 weeks) were included in the study, of which 42 (18.3%) demonstrated intra-abdominal calcifications, and 16/42 (38.1%) were mentioned in the radiology reports. Most calcifications were found to be within the lumen of the gastrointestinal tract, and in the left upper quadrant of the abdomen. There was no statistical difference between identifiable causes for fetal loss at autopsy in cases with and without calcification at PMSS (59.5% versus 58.5% respectively, *p*=0.904). Nevertheless, where calcification and a cause for fetal loss were found, the aetiology was more likely to be due a fetal rather than placental issue.

**CONCLUSION:**

The presence of internal calcifications on PMSS was not associated with an increased likelihood of explainable fetal loss or particular diagnosis at autopsy.

## Highlights

• Fetal calcifications on post-mortem skeletal surveys are not significantly associated with causes of fetal loss.
• When present, internal calcifications were usually seen in lower gestational aged fetuses, intra-abdominal in location, with the majority being intraluminal.
• The majority of radiologists do not report internal fetal calcifications on radiographs, without significant consequences for final outcome at autopsy.

## Introduction

Perinatal post-mortem skeletal surveys (PMSS, also known as a skeletal radiographs or a “babygram”) form part of a comprehensive perinatal autopsy examination.^1^ The radiographic assessment of the whole fetus provides an overview of skeletal development, presence of underlying inheritable bone disorders, and can be utilised to provide an estimate of gestational age by measurement of long-bone lengths.^2^

Intra-thoracic and abdominal calcifications can also be readily appreciated on skeletal surveys^3^; however, there is little information regarding their significance within the radiological literature. From autopsy data, several studies have reported an association between hepatic calcification with chromosomal abnormalities and transplacental infections,^4^, ^5^, ^6^, ^7^ and there are emerging data that calcifications within the bowel, soft tissues, and myocardium may also be a marker for underlying genetic disorders.^8^^,^^9^ Furthermore, individual case reports have suggested that meconium calcification may be a feature of underlying congenital anorectal malformations, possibly from the intermixing of urine with meconium via a recto-vesical fistula.^10^

Although several publications have reported a low diagnostic value and yield in performing routine PMSS (of approximately 0.27%^11^ to 5.3%^12^), these outcomes have been primarily focussed on the detection of severe musculoskeletal abnormalities (e.g., skeletal dysplasias), many of which were diagnosed antenatally. The significance of intra-thoracic and abdominal calcifications have not been assessed and, despite autopsy data highlighting their importance, many radiologists fail to report their presence,^3^ and when they do it is misinterpreted as meconium peritonitis (given the more common association in live children^13^) despite its rarity in the setting of perinatal deaths.^14^^,^^15^

The primary objective of the present study was therefore to determine whether the presence of internal calcifications (detected on perinatal PMSS) are associated with identifiable causes for fetal loss, and whether these findings have significance to radiologists during PMSS reporting. If internal calcifications are a marker of abnormality, then it could potentially guide the need for further tissue sampling for genetic or metabolic analysis at autopsy.

## Materials and methods

### Study cohort

Ethical approval was not required for this study as it was performed as part of a retrospective audit on data imaging quality and imaging features.

A retrospective review of the institution's radiology information system (RIS) was conducted for all perinatal PMSS performed over a 6-month period (October 2018 to April 2019). All cases had signed parental consent forms for either a full “invasive autopsy” or an “imaging”-based autopsy (e.g., with post-mortem magnetic resonance imaging [MRI] or micro-computed tomography [CT]) for assessment of structural congenital abnormalities.

Forensic or coronial cases were excluded, as the results of these autopsies are not made public routinely, and medicolegal proceedings may be ongoing. No inclusion or exclusion criteria were set regarding gestational age, mode of pregnancy loss or post-mortem interval (PMI; i.e., time between delivery and the imaging studies and autopsy). Demographic details obtained from the clinical records for each patient included their date of birth, date of death, gender, gestational age, crown–rump length and post-mortem weight.

### PMSS imaging

All PMSS imaging was performed using a dedicated Hewlett Packard 43855B Faxitron apparatus (Faxitron Bioptics LLC, Tucson, AZ, USA), at 3 mAs using a low kilovolt technique. Two images comprising a full-body frontal and lateral view projection (including axial skeleton and extremities) were acquired for all cases. The radiography was always performed prior to the autopsy or any further post-mortem cross-sectional imaging. At Great Ormond Street Hospital, all radiographs are routinely reported by one of seven specialist consultant paediatric radiologists, with >10 years of radiological experience for the assessment of inheritable bone disorders and gestational age estimation.

### Retrospective image analysis

All PMSS images and reports were re-reviewed retrospectively by one independent reader (S.C.S.) with 10 years of radiological experience (5 years of paediatric experience, of which 3 years were of paediatric post-mortem experience). The reader assessed the skeletal surveys for^1^ the presence and location of internal calcifications (i.e., intracranial, thoracic, abdominal)^2^; whether the calcification was mentioned in the original radiology report; and^3^ if so, whether any explanation was provided for this.

### Autopsy correlation

The type of autopsy conducted was based on parental choice as detailed in the autopsy consent form. Where a full “invasive” autopsy was performed, this was conducted by one of four paediatric specialist pathologists with >10 years of experience, according to Royal College of Pathologists' national guidelines.^16^^,^^17^ Routine genetic analysis is not performed as part of the perinatal autopsy.

Where an “imaging”-based autopsy was performed, this was conducted according to local departmental protocols, which have been previously published for post-mortem MRI and micro-CT imaging.^18^^,^^19^ Cases weighing >500 g are usually referred for post-mortem MRI (PMMR)^20^ and those smaller are referred for iodinated micro-CT imaging. Cross-sectional imaging reports were all interpreted by a paediatric radiologist with an interest in post-mortem imaging, with >15 years of radiological experience (O.J.A.).

Autopsy reports (including radiology reports, antenatal history, placental histopathology and external examination) were reviewed retrospectively for the cohort by a separate independent reader (C.R.), a board-certified radiologist with 7 years of experience who had not reviewed or analysed any of the original skeletal surveys or post-mortem cross-sectional imaging, in order to reduce bias.

All autopsy reports were reviewed for information regarding^1^ identification and location of internal calcification at fetal autopsy or cross-sectional imaging^2^; main diagnosis or presumed cause of fetal loss (structural anomalies or antenatal genetic analysis); and^3^ for abnormal placental histopathology.

### Statistical analysis

The frequencies and percentages of significant autopsy findings and causes for fetal loss in perinatal deaths with and without internal calcifications were compared. Statistical analysis was performed using Student's *t*-test for continuous, normally distributed data (e.g., gestational age, weight, crown rump length) and two-tailed Fisher's test was used for differences in categorical data.

A chi-squared test was used for differences in proportions between the explained and unexplained causes for fetal loss between the two groups. Sensitivity, specificity, and positive (PPVs) and negative predictive values (NPVs) for whether the presence of internal calcification could detect an explainable cause for fetal loss were also calculated. Analysis was performed using SPSS 18.0 for Windows (SPSS, Chicago, IL, USA). A *p-*value of <0.05 was deemed statistically significant.

## Results

### Demographics

Over the 6-month retrospective review period (18 October 2018 to 18 April 2019), 301 PMSS were performed, of which 71 were excluded due to childhood or infant deaths (i.e., non-perinatal deaths). There were no perinatal forensic or coronial cases. This resulted in a final dataset of 230 cases for review.

**Figure 1:**
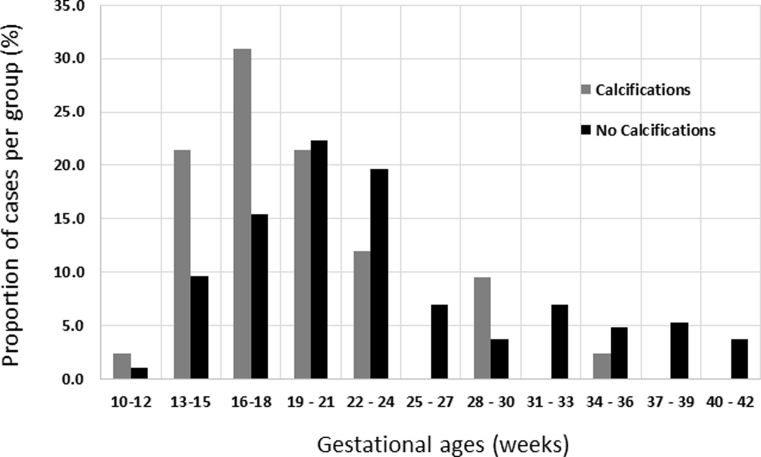
Graph illustrating the proportion (%) of fetuses with and without internal calcifications identified in the present cohort by gestational age. There was a significant difference between the two groups, with calcifications seen more commonly in lower gestational ages.

The case demographics are outlined in Table 1. A breakdown of cases that underwent termination of pregnancy and the indications for these are provided in the Electronic Supplementary Material, Table S1. There were statistically significant differences between the two groups, with those demonstrating internal calcifications on PMSS having a lower gestational age, crown–rump length, post-mortem weight, and also, a slightly longer PMI (time between death/delivery and autopsy). The proportion of cases within each group (i.e., with and without internal calcifications) at differing gestational ages are demonstrated in Fig 1. There were more stillborn cases and more cases that underwent a “full/invasive autopsy” in the “no calcification” group.

**Table 1:** Case demographics for study cohort.

	Total cases (*n* = 230)	Internal calcifications (*n* = 42)	No internal calcifications (*n* = 188)	*p*-Value
Median gestational age, weeks (range)	21 (12–41)	18 (12–35)	22 (12–41)	<0.001^a^
Median crown rump length, cm (range)	17.4 (3.2–40)	11.5 (6.1–35.4)	18 (3.2–40)	<0.001^a^
Median post-mortem weight, g (range)	271.5 (8–4126)	76.5 (8–3335.7)	300 (8–4126)	<0.001^a^
Post-mortem interval, days (range)	7 (1–24)	10 (3–24)	7^1^, ^2^, ^3^, ^4^, ^5^, ^6^, ^7^, ^8^, ^9^, ^10^, ^11^, ^12^, ^13^, ^14^, ^15^, ^16^, ^17^, ^18^, ^19^, ^20^, ^21^, ^22^, ^23^, ^24^	<0.05^a^
Gender (%)				
Male	103 (44.8)	18 (42.9)	85 (45.2)	NS
Female	117 (50.9)	19 (45.2)	98 (52.1)	NS
Unspecified	10 (4.3)	5 (11.9)	5 (2.7)	<0.05^a^
Mode of delivery (%)				
Termination	67 (29.1)	17 (40.5)	50 (26.6)	NS
Miscarriage	103 (44.8)	20 (47.6)	83 (44.1)	NS
Stillborn/intrauterine death	60 (26.1)	5 (11.9)	55 (29.3)	<0.05^a^
Autopsy type (%)				
Imaging only	100 (43.5)	25 (59.5)	75 (39.9)	<0.05^a^
Limited	1 (0.4)	0	1 (0.5)	NS
Full/invasive	129 (56.1)	17 (40.5)	112 (59.6)	<0.05^a^

Post-mortem interval denotes the time between death/delivery and autopsy (regardless of whether this was imaging or conventional autopsy). *p*-Values calculated using unpaired *t*-test for continuous data, and Fisher's test for categorical data.

NS, not significant.

a Denotes statistical significance.

### Location of calcifications on PMSS

**Figure 2:**
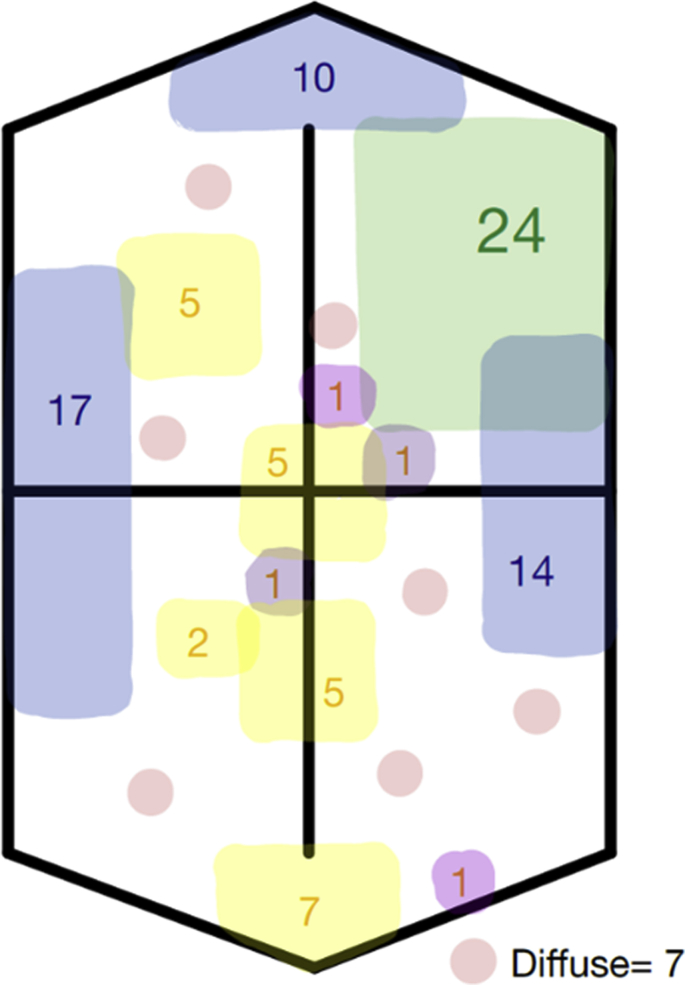
Diagrammatic representation for the various locations of internal calcifications (by percentage, %) seen within the present cohort (*n* = 42).

In total, only 42 (42/230, 18.3%) cases demonstrated internal calcification on the PMSS. In all of these cases, the calcification was intra-abdominal. No foci of intracranial or intrathoracic calcification were seen. The distribution of the calcifications is displayed diagrammatically in Fig 2. The majority of intra-abdominal calcifications were in the left upper quadrant.

### Location of calcification at autopsy

In 129/230 (56.1%) cases, conventional (invasive) autopsy was performed (of which 18 had intra-abdominal calcifications on skeletal survey). For these 18 fetuses, the presence of calcification was not mentioned in any of the final autopsy reports.

**Figure 3:**
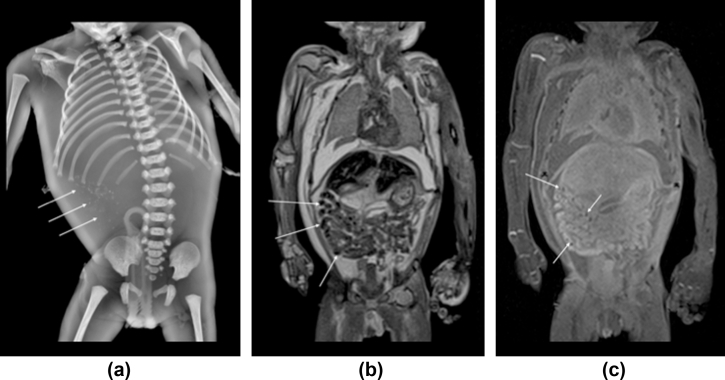
A 29-week-old stillborn fetus, found to have duodenal atresia and hypoplastic aortic arch at autopsy. The frontal PMSS (a) demonstrates multiple linear calcific densities in the right hemiabdomen. The subsequent post-mortem MRI images, presented in coronal section on the (b) T2-and (c) T1-weighted sequences demonstrate low signal intraluminal material (arrows), consistent with calcified meconium.

**Figure 4:**
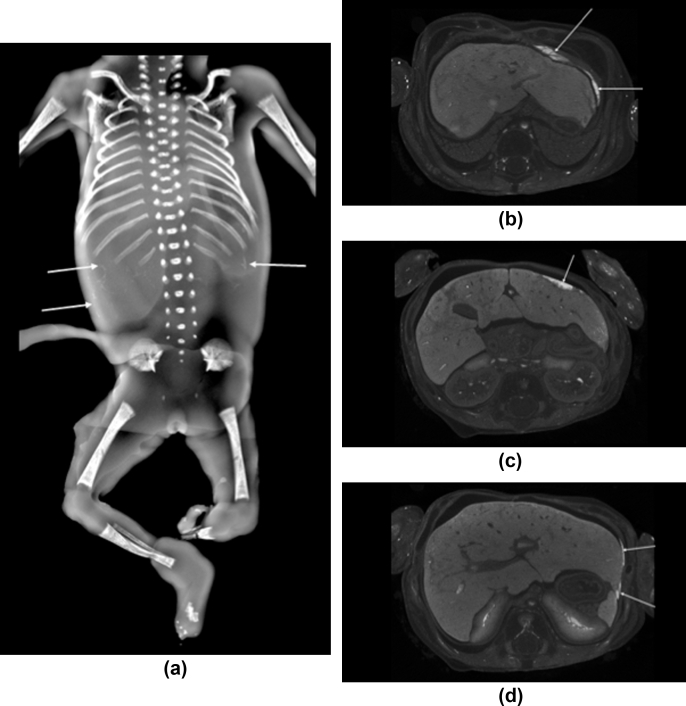
An 18-week gestational age fetus with amniotic band syndrome. Frontal view of the skeletal survey (a) demonstrates punctate flecks of upper abdominal calcification (white arrows). (b)Axial post-mortem micro-CT imaging of the upper abdomen, acquired at 40 μm resolution, demonstrates calcification along the left hemidiaphragm, surface of the left lobe of the liver and (c) within the right hepatic lobe as well as (d) along the subcapsular region of the left lobe of the liver (white arrows).

**Figure 5:**
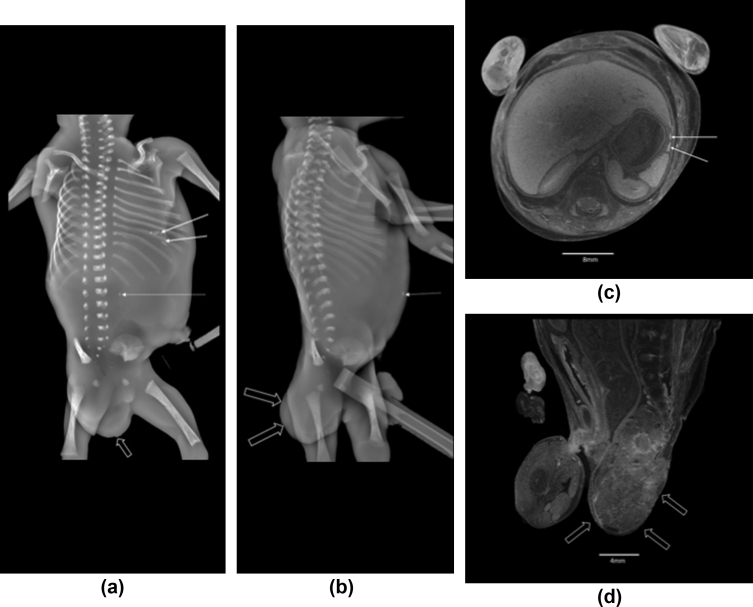
A 16-week gestational aged fetus with sacro-coccygeal teratoma. The frontal (a) and lateral (b) PMSS radiographs reveal left upper quadrant calcification (solid white arrow) and a midline focus of calcification (dotted arrow), the latter felt to represent artefact on surface of fetal body. The large soft-tissue mass representing the teratoma (open, unfilled arrow) does not contain any internal calcification on PMSS. At subsequent micro-CT, acquired at 75 μm resolution (c), the left upper quadrant calcification was intra-peritoneal. A sagittal section of the pelvis on micro-CT, acquired at 18 μm resolution (d) demonstrates the presacral mass. There was no internal calcification, the high density within the lesion represents pooling of contrast media.

**Figure 6:**
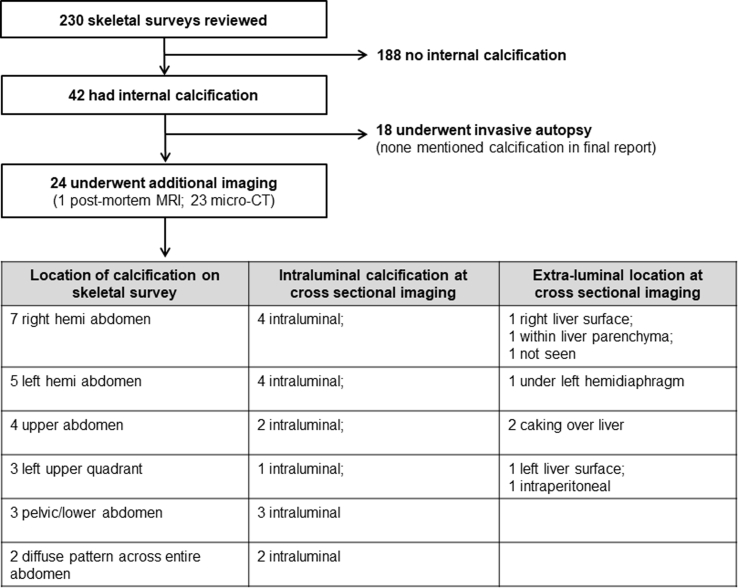
Flowchart demonstrating the total number of cases reviewed, and those that were subsequently found to have internal calcifications with their corresponding location, as seen on further post-mortem cross sectional imaging.

In 100/230 (43.5%) cases, a non-invasive, imaging autopsy was performed using either high-resolution micro-CT or post-mortem MRI (of which 24/100 had intra-abdominal calcifications on skeletal survey). For these 24 fetuses, the calcification was clearly identified in 96% (23/24) of cases on the cross-sectional imaging. Subtle intra-abdominal calcification was difficult to locate in one case, possibly due to maceration. The anatomical location at cross-sectional imaging of the calcification, when seen, was mostly intraluminal (16/24, 66.7%) within small bowel loops (Fig 3). In 7/24 (29.2%) cases, the calcification was either intraperitoneal, intrahepatic, or covering the surface of the liver (Figure 4, Figure 5). A flowchart (Fig 6) summarises the correlation between location of calcification at PMSS and subsequent cross-sectional findings.

A limited autopsy was performed in only one case (without internal calcifications), with post-mortem MRI and a limited dissection of the heart and lungs, but not of the abdomen or brain.

### Comparison of calcification versus non-calcification groups

A cause for fetal loss was provided at autopsy in 25/42 (59.5%) cases with intra-abdominal calcification compared to 110/188 (58.5%) cases without calcification. A chi-squared test for differences in proportions between the explained causes for fetal death was not statistically significant between the two groups (*p*=0.904; Table 2). If using internal calcification as a marker for “an explainable cause for fetal loss”, the diagnostic accuracy rates would be poor. These include a sensitivity of 59.5% (43.4–74.4%); specificity of 41.5% (34.3–48.9%), PPV of 18.5% (14.7–23.1%), NPV of 82.1% (75.3–87.3%), and concordance of 44.8% (38.2–51.5%).

**Table 2:** Cause of death between different fetal groups with and without intra-abdominal calcifications at skeletal survey.

	Total cases (*n* = 230)	Internal calcifications (*n* = 42)	No internal calcifications (*n* = 188)	*p*-Value
Cause of death (%)	135 (58.7)	25 (59.5)	110 (58.5)	NS
Fetal anomalies	67 (29.1)	18 (42.9)	49 (26.1)	<0.05^a^
Placental anomalies	68 (29.6)	7 (16.7)	61 (32.4)	<0.05^a^
Unexplained cause (%)	95 (41.3)	17 (40.5)	78 (41.5)	NS

NS, not significant.

a Denotes statistical significance.

Within the “internal calcification” group, where a cause for fetal loss was found, there was a statistically significant relationship between calcification and a fetal cause for the loss (*p*=0.03) with relative risk of 1.29 compared to a placental cause for fetal loss (*p*=0.043) with relative risk of 0.83. Therefore, if intra-abdominal calcifications were present on post-mortem imaging and a cause of death/fetal loss was found, this would be 29% more likely to be from a fetal abnormality rather than a placental abnormality; however, given the wide range of underlying fetal anomalies in the present cohort and locations of calcification, it was not possible to attribute any one particular anomaly to specific locations and patterns of intra-abdominal calcification.Where internal calcifications were present, Table 3 provides the location of the calcifications and causes for fetal loss (including locations for calcifications were cause was unexplained). In the cases without any internal calcifications, Table 4 provides the reported causes for fetal loss at autopsy.

**Table 3:** Location of internal calcifications in those with placental and fetal causes for the fetal loss (*n* = 25).

Location of calcification on skeletal survey	Cause of fetal loss/main diagnosis (or no. of cases if unexplained)
Unexplained causes (*n* = 17)	
Diffuse (throughout abdomen)	1
Left upper quadrant	2
Left hemiabdomen	3
Upper abdomen	3
Right hemiabdomen	4
Lower abdomen/pelvis	4
Fetal causes (*n* = 18)	
Left upper quadrant	Sacrococcygeal teratoma
	Fetal growth restriction
	Nuchal thickening, collapsed stomach, left sided sub diaphragmatic cyst, absent left kidney, narrowing of aorta Antenatally counselled for increased risk of Down's, trisomy 18, and 13
	Neural tube defect
	Fetal hydrops, cause unknown
	Fetal hydrops, cause unknown
	Thanatophoric dysplasia
Left hemiabdomen	Possible triploidy raised (no genetic testing): cleft palate, polydactyly, fetal growth restriction
	Fetal hydrops, cause unknown
Upper abdomen	Bowed right femur and short right tibia: likely isolated insult, not a skeletal dysplasia
	Amniotic band syndrome
Right hemiabdomen	IUGR, duodenal atresia, hypoplastic aorta, antenatally diagnosed unbalanced rearrangement of 12p19q genetic defect
	Facial anomalies and renal cysts, suggestive of trisomy 13
	Fetal hydrops
	Congenital diaphragmatic hernia
	Renal dysplasia and limb anomalies
Lower abdomen/pelvis	Neural tube defect
	Hydrops, bilateral renal agenesis with cardiomyopathy
Placental causes (*n* = 7)	
Diffuse (throughout abdomen)	Multiple villous infarctions
	Chronic villitis with cytomegalovirus positive microbiology
Left upper quadrant	Ascending maternal genital infection
	Delayed villous maturation of the placenta
Left hemiabdomen	Maternal vascular malperfusion
Upper abdomen	Maternal vascular malperfusion
Right hemiabdomen	Chronic histiocytic intervillositis

Cases where the final outcome was “unexplained fetal loss”, who also had internal calcification on skeletal survey were described in this table also.

**Table 4:** Cases that did not have calcification on skeletal survey and causes of fetal loss (*n* = 188).

Cause of fetal loss/main diagnosis	No. of cases (%)
Unexplained fetal loss	78 (41.4)
Fetal causes (*n* = 49, 26.1%)	
Complex congenital intracranial anomalies	12 (6.4)
Complex congenital cardiac anomalies	8 (4.3)
Trisomy 18 (clinically suspected from structural anomalies)	6 (3.2)
Neural tube defects	4 (2.1)
Skeletal dysplasias	4 (2.1)
Genitourinary abnormalities	3 (1.6)
Amniotic band syndrome	2 (1.1)
Arthrogryposis multiplex congenital	2 (1.1)
Fetal hydrops	1 (0.5)
Severe prematurity	1 (0.5)
VACTERL sequence	1 (0.5)
Acute fetal blood loss	1 (0.5)
Multisystem anomalies: exomphalos, ventriculomegaly, anal atresia	1 (0.5)
Trisomy 21 (antenatally detected)	1 (0.5)
Potter's sequence	1 (0.5)
Noonan's syndrome	1 (0.5)
Placental causes (*n* = 61, 32.4%)	
Ascending maternal genital infection/chorioamnionitis	40 (21.3)
Maternal/placental vascular insufficiency	12 (6.4)
Twin to twin transfusion syndrome	4 (2.1)
Retroplacental haemorrhage	2 (1.1)
Delayed villous maturation of the placenta	1 (0.5)
Chorionic haemosiderosis	1 (0.5)
Fetal thrombotic vasculopathy	1 (0.5)

VACTERL, vertebral defects, anal atresia, cardiac defects, tracheo-oesophageal fistula, renal anomalies, and limb abnormalities.

None of the fetuses demonstrating internal calcifications were reported as having meconium peritonitis. Although genetic testing was not performed routinely in perinatal autopsies, 3/42 (7.1%) cases with internal calcifications versus 7/188 (3.7%) without calcifications were suspected of having an underlying genetic aneuploidy (base on antenatal investigations or pattern of structural anatomical anomalies at autopsy).

### Radiologists reporting of calcifications

In 16/42 (38.1%) cases, the reporting radiologist mentioned the presence of the intra-abdominal calcifications, and in only 9/16 (56.3%) cases was a description or explanation ascribed to this finding (seven were reported as calcified intraluminal meconium of no significance and two were attributed to intrauterine perforation).

At autopsy, for the seven cases reported as “intraluminal calcification of no significance”, there were four unexplained fetal losses, one had a neural tube defect, one had fetal hydrops of unknown aetiology, one had a known genetic abnormality detected antenatally (12p19q unbalanced rearrangement) with duodenal atresia and a hypoplastic aortic arch at autopsy. The two cases reported as “meconium perforation” did not have evidence of perforation; one case was reported an unexplained fetal loss at autopsy, the other had profound fetal growth restriction. In the seven other cases where calcification was reported on the radiograph, but an explanation for this was not given by the radiologist, two were unexplained fetal losses and the remaining five demonstrated fetal anomalies at autopsy (two were from fetal hydrops of unknown aetiology; one congenital diaphragmatic hernia; one renal dysplasia and limb anomalies; one absent left kidney, sub-diaphragmatic cyst, and hypoplastic aorta).

## Discussion

This study shows that fetal calcification on PMSS is not significantly associated with underlying fetal or placental abnormalities attributable to the fetal loss. The predictive value of intra-abdominal calcification on PMSS is poor. Where present, calcification was intra-abdominal and mostly intraluminal. Where a cause for fetal demise was found, then the calcification was more likely due to a fetal (than placental) aetiology. Most radiologists did not mention the calcification, and this was of little clinical consequence.

To the authors' knowledge, other published work on post-mortem fetal calcification has not included their presence on skeletal radiographs, or their implications for the reporting radiologist. Although it is prudent to report all pertinent findings on plain radiography, the reporting of fetal calcification was not found to be useful in helping associate a particular cause for the fetal loss, and interestingly, none of the pathologists routinely included internal calcification at autopsy, probably due to it being mostly intra-luminal or too subtle.

When compared to the published autopsy data on fetal calcifications, the present data support the findings that calcifications are more likely to be found in fetuses of a lower gestational age group (and thus smaller crown rump length and post-mortem weights, as the present study found). The median gestational age for fetuses with calcifications was 18 weeks gestation (compared to 22 weeks gestation for those without) in the present work. Sahlin *et al.* found that the highest proportion of internal calcifications were seen in fetuses of 13–15 weeks gestation (>10% of cases) compared to ≤5% in cases of ≥19 weeks gestation.^9^ The reasons for this remain unclear.

In contrast to the autopsy literature, the locations and prevalence of fetal calcifications on PMSS differed. A higher prevalence was found than other series, with 18.3% of all fetuses at PMSS demonstrating calcifications over a 6-month period, with the majority located within bowel loops. One autopsy study reported an overall proportion of 5.3% of fetuses over a 9-year study period having internal calcifications, with 57% located in a perihepatic or intrahepatic location with the second most common site being cardiac (13%), followed by bowel (9%).^9^ Further publications have focussed solely on fetal hepatic calcifications (presumably given their commoner occurrence at autopsy), and report a prevalence between 2.3%^4^ and 4.2%^7^ in large fetal databases (>500 cases each). One possible reason for this could be due to the variability in assessment of intraluminal contents at autopsy.

Conversely, studies from antenatal ultrasound findings of “echogenic bowel loops” (which may cause post-mortem intraluminal calcification) have been reported as being relatively common, occurring in approximately 1% of all pregnancies.^21^ Although previous work published in the 1990s described a strong association between echogenic bowel and underlying chromosomal disorders, mainly trisomies,^22^, ^23^, ^24^ it has been reported more recently that this may not a risk factor where the echogenic bowel is an isolated finding.^25^ In one cohort of 409 fetuses with echogenic bowel on antenatal ultrasound, 82.6% were not found to have any congenital anomalies or genetic disorders with further investigations and clinical follow-up after delivery.^21^ It is therefore not that surprising that within the present cohort, given the higher frequency of intraluminal calcifications over other locations, no significant differences were found regarding causes for fetal loss; however, it is acknowledged that the present population differs from those undergoing routine antenatal imaging, which resulted in mostly successful pregnancies.

In terms of causes for fetal losses, previous autopsy studies found a higher incidence of aneuploidy where fetal calcifications were present.^4^^,^^6^^,^^7^^,^^9^ One major limitation of the present study is the lack of routine genetic testing during perinatal autopsy, precluding the analysis of this feature. From reviewing the antenatal history and conclusions in the autopsy reports, proportionally twice as many suspected aneuploidy cases were present in the fetal calcification group versus the non-calcification group (3/42 [7.1%] versus 7/188 [3.7%]). Nevertheless, these numbers are small and it is hard to draw any firm conclusions when not all cases underwent genetic analysis. Interestingly, although meconium peritonitis or intra-uterine perforation was deemed the cause for fetal calcification in two of the reported cases by the radiologist, there were no cases in the entire cohort with this finding (and apart from one case with an unbalanced rearrangement of 12p19q with duodenal atresia, no other cases with intestinal atresias that may have resulted in perforation). One possible explanation for the intraperitoneal calcification may relate to calcified blood products. It is well known that the fetal liver is enlarged and highly vascular *in utero*, and also that injury to this organ during extraction or delivery can cause subcapsular haematomas and bleeding.^26^ Disruption and damage to this organ may be made more susceptible with concomitant sepsis, coagulopathies, hypoxia and maternal diseases, which were common in the present cohort. Although there is no doubt regarding the diagnosis of meconium peritonitis, particularly when identified on live neonatal radiographs, its occurrence is rare, and even when present, would be unlikely to be the cause for fetal demise.^27^^,^^28^ The literature does not offer any explanation for the underlying mechanism for intraluminal calcified meconium, in the absence of any structural or anorectal malformation; however, one interesting result from the present study was the slightly longer PMI (i.e. time between delivery and imaging) for fetuses with internal calcifications. This raises the possibility that some of the calcification could be in part, due to calcification developing in the post-mortem period. Further studies on this will need to be performed to confirm or refute this hypothesis.

As with all studies, there were several limitations. First, the precise location of all calcification identified on PMSS could not be confirmed due to parental consent for or against cross-sectional imaging or autopsy. There was variability between pathologists' identification of calcification at autopsy. Not all cases underwent genetic analysis, and therefore, a possible association between internal calcifications and underlying chromosomal abnormalities could not be confirmed.

Secondly, as a tertiary-referral centre for specialist paediatric pathology work, the caseload may not be representative of the wider community. The rate of “unexplained” fetal causes of death in the present study, at approximately 40%, is comparable to published data from other similar centres.^29^, ^30^, ^31^

Finally, although over 200 radiographs were reviewed, the study was not a powered study, and therefore, the sample size may have been too small. As there was almost an identical percentage of unexplained causes of death between the two cohorts, this sample is representative. There were no situations where the reporting (or lack thereof) of internal calcifications by the radiologist could have changed conclusions drawn at autopsy regarding fetal or placental anomalies.

With an increase in parental demand for less invasive autopsies, and as knowledge regarding the genetic basis of diseases increases,^32^^,^^33^ future work may include a larger prospective perinatal cohort with routine genetic testing and post-mortem cross-sectional imaging to better understand the significance of calcification on imaging. It could possibly also reveal the pathogenesis and mechanisms behind the presence of this feature and whether this does hold any merit as a biomarker for underlying disease. In terms of identification of structural fetal anomalies and placental disease, this marker was not helpful in the present study.

In conclusion, fetal calcification on PMSS is not significantly associated with underlying fetal or placental abnormalities and is often unreported. As most radiologists do not mention its presence, there may be limited value in its identification in future.

## Declaration of interests

The authors declare no conflict of interest.
