# Supplementary material for: The significance of internal calcifications on perinatal post-mortem radiographs
Source: Clin Radiol. 2020 Jul;75(7):561.e25–34. doi: 10.1016/j.crad.2020.03.007 (PMC7296345; doi:10.1016/j.crad.2020.03.007)
Supplement: Multimedia component 1 [file mmc1.docx]

**APPENDIX**

**Table A1. Indications and mode of termination of pregnancy (TOP), divided by presence of internal calcifications on skeletal survey.** Of the 67/230(29.1%) fetuses in our study group that underwent a termination of pregnancy, the mode of termination and indication for TOP are provided below. Where the method for termination was not included in the autopsy report, the method has been classified as ‘unknown’.

|  | **Internal calcifications**  **(n = 17)** | **No internal calcifications**  **(n = 50)** |
| --- | --- | --- |
| ***Mode of TOP*** | | |
| *Intracardiac injection*  *Induction of labour*  *Unknown* | 2 (11.8%)  8 (47.1%)  7 (41.2%) | 13 (26.0%)  15 (30.0%)  22 (44.0%) |
| ***Indications for TOP*** | | |
| *Neurological anomalies* | 3 (17.6%)  *(1 neural tube defect,*  *1 sacrococcygeal teratoma,*  *1 caudal regression syndrome)* | 17 (34%)  *(3 ventriculomegaly,*  *4 cerebellar hypoplasia,*  *4 absent corpus callosum,*  *4 neural tube defect,*  *1 polymicrogyria,*  *1 vein of Galen malformation)* |
| *Cardiac anomalies* | 1 (5.9%)  *(hypoplastic pulmonary valve)* | 3 (6%)  *(2 double outlet right ventricle,*  *1 polyvalvular dysplasia with bilateral superior vena cavae)* |
| *Abdominal anomalies* | 0 | 3 (6%)  *(1 body stalk anomaly,*  *2 exomphalos)* |
| *Genitourinary anomalies* | 1 (5.9%)  *(bilateral renal agenesis)* | 3 (6%)  *(2 bilateral renal agenesis,*  *1 bladder outlet obstruction)* |
| *Musculoskeletal anomalies* | 3 (17.6%)  *(1 thanatophoric dysplasia,*  *1 amniotic band syndrome,*  *1 shortened and bowed femora)* | 6 (12%)  *(1 Fryn’s syndrome,*  *2 arthrogryposis multiplex,*  *1 thanatophoric dysplasia,*  *1 osteogenesis imperfect,*  *1 VACTERL spectrum)* |
| *Fetal hydrops* | 4 (23.5%) | 4 (8%) |
| *Increased risk of trisomy* | 3 (17.6%) | 6 (12%) |
| *Fetal growth restriction* | 2 (11.8%) | 1 (2%) |
| *Maternal/obstetric issues* | 0 | 7 (14%)  *(1 premature rupture of membranes, 2 anhydramnios,*  *4 chorioamnionitis)* |
